# Exosomes in Gliomas: Biogenesis, Isolation, and Preliminary Applications in Nanomedicine

**DOI:** 10.3390/ph13100319

**Published:** 2020-10-19

**Authors:** Eugenia Romano, Paolo Antonio Netti, Enza Torino

**Affiliations:** 1 Department of Chemical, Materials Engineering & Industrial Production, University of Naples Federico II, Piazzale Tecchio 80, 80125 Naples, Italy; eugenia.romano@unina.it (E.R.); paoloantonio.netti@unina.it (P.A.N.); 2 Interdisciplinary Research Center on Biomaterials, CRIB, Piazzale Tecchio 80, 80125 Naples, Italy; 3 Center for Advanced Biomaterials for Health Care, CABHC, Istituto Italiano di Tecnologia, IIT@CRIB, Largo Barsanti e Matteucci 53, 80125 Naples, Italy

**Keywords:** extracellular vesicles, miRNA, biomarkers, therapeutics, tumor microenvironment, glioma, brain diseases, intercellular communication

## Abstract

Exosomes are phospholipid-based particles endogenously produced by both normal and tumor cells. Initially identified as a pathway for shuttling cellular waste, for a long time they were thought to act as “garbage bags”, and only in the past few years have they emerged as a promising drug delivery system. In this review, we provide an overview of the knowledge about exosome architecture and biogenesis and the recent progress in isolation methods. Furthermore, we describe the mechanisms involved in both extra- and intracellular communication with a focus on glioma brain tumors. Glioma is considered a rare disease and is the most prominent aggressive brain malignancy. How exosomes target glial tumoral cells in vivo remains largely unknown. However, they are able to influence numerous physio-pathological aspects. Here, we discuss the role they play in this heterogeneous and complex microenvironment and their potential applications.

## 1. Introduction

More than 40 years ago, vesicle structures, similar to “cytoplasmic fragments” physiologically released, were identified in the cellular matrix. Their peculiarity was their ability to contain various materials, including ribosomes, which are involved in several pathological and physiological activities. After being initially considered as part of the lysosomal degradation pathway [1] through which the cell ejected its waste, they are now recognized as important messengers involved in proximal and distal intercellular communication. These systems were defined as extracellular vesicles (EVs), and they include a wide variety of vesicles (from 30 nm to 5 μm) released from the plasma membrane (PM) of many different cell types into several bodily fluids, including plasma, milk, saliva, sweat, tears, semen, and urine [2,3].

All EVs present a lipid bilayer membrane that surrounds a pool of genetic material, cytosolic proteins, or cellular debris [4]. However, they significantly differ in terms of size, biogenesis, mechanisms, and function. For this reason, they are generally categorized into three subtypes: exosomes, ectosomes, or Shedding MicroVesicles (SMVs) and apoptotic bodies [5,6].

Exosomes have sizes ranging between 30 and 150 nm and represent a homogenous population of EVs released from cells when multivesicular bodies (MVBs) are fused with the membrane through inward budding in a highly regulated process [7].

In contrast, SMVs are a more heterogeneous population of EVs, with a size ranging from 50 nm to 1 μm, which are formed under specific physiological stimuli, such as calcium-dependent signaling, by the budding and shedding of PM [5,6].

Consequently, exosomes and SMVs are currently believed to have endosomal and PM origins, respectively [8].

Finally, apoptotic bodies, composed of cytoplasmic organelles and fragmented nuclei, are EVs 1–5 μm in diameter. They are formed when a cell is dying via apoptosis. After the disruption of PM, the cytoplasmic content is divided into different membrane-enclosed vesicles [9].

Among these membrane vesicles, the role of exosomes in cancer research has been rapidly growing over the last two decades. Cancer cells release a high number of exosomes containing many functional biomolecules in the extracellular space. EVs transfer proteins, receptors, and small RNAs that regulate both physiological and pathological processes. Moreover, the lipid bilayer membrane protects the exosome cargo from degradation in the bloodstream, allowing crossing different physiological barriers, such as the Blood–Brain Barrier (BBB) [10].

BBB is one of the most complex and selective barriers in the human organism. Its principal role is to preserve the homeostasis of the central nervous system and protect the brain parenchyma against the invasion of inflammatory mediators, which may interrupt its critical function [11]. The BBB, together with pericytes, perivascular astrocytes, microglia, and neurons, forms a functional unit called the neurovascular unit. Interestingly, EVs regulate the communication between cells in short or long distances within the neurovascular unit [12]. Furthermore, exosome cargo such as miRNAs, proteins, and other physiological compounds reflect different brain disease progression stages, allowing their use as a “window to the brain” [11].

As natural carrier systems, EVs present low immunogenicity, low toxicity, stability in the bloodstream, and efficient cell uptake due to their endogenous cellular tropism [13]. Their ability to mediate intercellular communication, especially in brain tumor progression, allows their use as a promising therapeutic and diagnostic tool [14,15,16,17,18,19,20,21], including the challenging treatment of gliomas [22,23,24,25,26,27]. Gliomas are the most frequent intrinsic tumors of the central nervous system and, by following the 2016 World Health Organization (WHO) update, encompass two principal subgroups: “nondiffuse gliomas”, showing a more circumscribed growth pattern (WHO grade I), and diffusely infiltrating gliomas (WHO grade II–IV), arising from glial cells or glial precursors [28]. Grade IV glioblastoma (GBM) is the most infiltrating, aggressive, and poorly treated brain tumor in adults. Despite the aggressive treatments used in GBM, such as surgical resection followed by radiotherapy and temozolomide (TMZ) therapy [29,30], limited drug penetration to the tumor sites and the rapid development of resistance to chemotherapy lead to poor prognosis. Thus, engineered exosomes could represent a valid alternative to conventional drug delivery systems. However, more studies are required to identify the specific receptors involved in the transport as well as the mechanism of interaction with target cells.

## 2. Biogenesis of Exosomes: A Spontaneous Formation

The biogenesis and release of exosomes in the extracellular space initiate an endocytic pathway at the PM [9]. Despite the fact that this event is not entirely clarified, it begins with the formation and progressive accumulation of intraluminal vesicles (ILVs) in MVBs. These late vesicles elude the lysosomal digestive system and, after the fusion with the PM, are finally secreted into the extracellular space [31].

**Figure 1:**
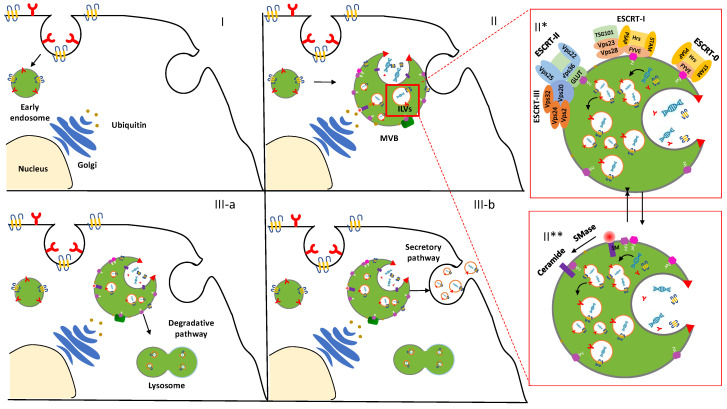
Biogenesis and secretion of exosomes. After the endocytosis of the plasma membrane, the transmembrane proteins are sorted into the vesicles that bud from the cellular membrane into “early endosomes” (I). The biogenesis of the exosome begins with the progressive formation and accumulation of ILVs inside MVBs (II). This process is mediated via an ESCRT-dependent (II*) and/or independent (II**) pathway. Then, the MVBs may follow a degradation pathway fusing with lysosomes or are destined to release the ILVs as exosomes to the extracellular space by exocytosis (III).

The physiological mechanism related to exosome formation and secretion is mediated via an Endosomal Sorting Complex Required for Transport (ESCRT)-dependent and/or ESCRT-independent pathway [32] (Figure 1).

## 3. ESCRT-Dependent Pathway

ESCRT machinery, conserved throughout eukaryotic and yeast cells, consists of four complexes—ESCRT-0, -I, -II, and -III—which act sequentially to bind and cluster ubiquitinylated proteins in the late endosome [33]. In particular, ESCRT-0 is involved in cargo clustering, ESCRT-I and ESCRT-II induce bud formation, and ESCRT-III drives vesicle scission. ILVs formation begins with the interaction between ESCRT-0—in particular, the two subunits hepatocyte growth-factor-regulated tyrosine kinase substrate (Hrs) and Signal Transducing Adaptor Molecule (STAM) in eukaryotic cells—and the region of the FYVE domain [34,35] of the endosomal lipid PhosphatidylInositol 3-Phosphate (PtdIns(3)P). Subsequently, the PSAP sequence (residues 348–351) in Hrs interacts with the Ubiquitin E2 Variant (UEV domains) of Tsg101 and Vps23 expressed in ESCRT-I, making possible both the formation of the ESCRT-0/ESCRT-I complex and the recruitment of ESCRT-I to endosomal membranes [36].

Then, the Vps36 subunit of ESCRT-II binds the ESCRT-I Vps28 C-terminal domain [37] through its GLUE N-terminal domain. ESCRT-III, the most ancient and preserved of the ESCRTs [38], consists of a core complex that contains the subunits Vps20, Vps32, Vps24, and Vps2, assembled in a highly ordered manner. However, these metastable subunits are present as inactive monomeric forms and, only after their conformational changes, the autoinhibition mechanism allows interactions with other ESCRT-III subunits. However, even though the activation of the assembly is not fully understood, it might happen in a directional order, so, one activated, the ESCRT-III subunit activates the next one [39,40]. The subunit that nucleates the ESCRT-III assembly on membranes is a N-terminally myristoylated subunit (Vps20). Vps20 from ESCRT-III binds to the Vps25 subunit of ESCRT-II. Finally, through the formation of this new complex, nascent ILVs results in the closing of the cargo-containing vesicle and the pinching off of the vesicles, even though how ESCRT-III oligomerization induces membrane curvature remains still elusive [39].

## 4. ESCRT-Independent Pathway

Conversely, a recent study of mammalian cells [41] showed, through the depletion of all four ESCRT key subunits, that, despite a dramatic alteration in the morphology of cellular components, early and late endosomes remain unaffected. Evidence of an ESCRT-independent pathway was shown also for melanosomes [42], lysosome-related organelles that contain melanin-producing enzymes and produce melanin. They are assembled within melanocytes, and their biogenesis involves a series of protein sorting and vesicular trafficking events: melanosomal protein Pmel17 is sorted into ILVs by a mechanism independent of lumenal determinants, and it is not affected by the functional inhibition of Hrs and ESCRT complexes [43]. These observations led to the conclusion that eukaryotes utilize mostly the established ESCRT system, as is already understood in yeast, and, probably, additional ESCRT-independent pathways to form ILVs. Indeed, an unconventional pathway seems to be driven by the presence of certain lipids such as ceramides, as confirmed in the membrane trafficking of the proteolipid content in the oligodendroglial murine cell line [44]. These data provided evidence for an alternative pathway depending on raft-based microdomains that may contain high concentrations of sphingomyelina (SM). Therefore, the hydrolytic removal of the phosphocholine moiety of SM by sphingomyelinases (SMases) induces ceramide formation that sequentially allows the coalescence of the small microdomains into larger ones. Finally, another protein that has been suggested to play a role in exosome formation is the Small Integral Membrane Protein of the Lysosome/late Endosome (SIMPLE, also called lipopolysaccharide-induced TNF factor, LITAF) [45]. After the transfection of cells with SIMPLE, an increased secretion of exosomes was observed, while SIMPLE mutation causes the loss of MVBs’ proper formation and exosome biogenesis [46].

Currently, it is possible to confirm that exosome biogenesis is a complex mechanism in which several compounds are involved. Structural and biochemical analyses of the upstream components and detailed studies of all the steps involved in the assembly and disassembly of the ESCRT complex contributed to its consideration as the main one implicated in EV biogenesis and clarified insights about EV formation and function. However, several studies have proved that biogenesis was not inhibited by the depletion of ESCRT subunits. This result increased evidence that other lipids and proteins play a key role in the membrane-invagination process.

Reinforcing this point, some features have to be considered. To date, it is legitimate to describe the presence of these two distinct processes as ESCRT-dependent or ESCRT-independent mechanisms. However, the activation of these alternative pathways is not fully elucidated and some aberrant ILV morphologies were observed while the early and late endosome remained differentiated [31]. Thus, we may hypothesize that the pathways are not entirely separated. They might work synergistically or influence each other. The cell type and/or cellular homeostasis could be an essential factor in controlling exosome secretion [47].

## 5. Isolation Techniques for the Collection of the Exosomes

To date, exosome purification is essentially based on size exclusion [48], polymeric precipitation [49,50], ultracentrifugation [51], and microfluidics [52,53] techniques. An ideal purification method should isolate exosomes from various biological sources in appreciable quantity and purity, but, due to their small size and heterogeneity, their isolation from interfering components, such as cellular debris and aggregated proteins, can be challenging.

## 6. Centrifugation-Based Isolation Techniques

Ultracentrifugation (UC)-based isolation is the most commonly used technique [51,54,55,56,57,58,59] according to a worldwide International Society for Extracellular Vesicles (ISEV) survey [60].

Currently, there is a standard protocol that includes several cleaning steps before the recovery of the exosomal sample [53], despite the fact that the final purity and concentration are extremely variable (Table 1).

Generally, 3 × 10^12^ particles per mg (p/mg) of protein has to be considered as a high purity value. Preparations with lower ratios, around three times lower (1 × 10^10^ p/mg), can be achieved readily by simple pellet and wash protocols. These are naturally inferior purifications containing significantly higher levels of contaminating proteins [61].

**Table 1:** Exosome quantification in term of particles and μg of protein per mL significantly changes according to the isolation method, cell source, and source amount. This highlights the absence of a standardized method to obtain a homogeneous sample.

Cell Source	Source Amount	Isolation Method	Exosome Yield	Reference
Non-Small-Lung Cancer (SK-MES-1)	150 mL Cell culture medium (CCM)	UC	1.3 × 10^12^ particles/mL	[51]
		UF	2 × 10^12^ particles/mL	
Human colon carcinoma (LIM186)	2 × 10^9^ cells	UC	375 μg protein	[54]
		Density gradient	75 μg protein	
		Immunoaffinity	195 μg protein	
Murine melanoma (B16BL6)	-	UC	6 × 10^11^ particles/mL	[55]
Mouse mammary carcinoma (4T1) Human mammary adenocarcinoma (MCF-7) Human prostate adenocarcinoma (PC3)	2 × 10^8^ cells	Density gradient	-	[56]
Macrophages (Raw 264.7)	2 × 10^8^ cells	UC	10^11^–10^12^ Exosome/flask–1 mg/mL total protein	[57]
Human primary GBM (U-87 MG)	280 mL CCM	UC	10^12^ particles/mL	[58]
Melanoma (B16F10)	72 mL CCM	UC	2.04 × 10^13^ ± 3.9 × 10^12^ p/mL 451.15 ± 71.5 μg/mL 4.52 × 10^10^ ± 1.26 × 10^10^ p/μg	[59]
Raw264.7	5 × 10^8^ cells	UC	1 mg	[57,62]
Mice Blood	10 mL CCM	UC	18 μg/mL protein 7.49 × 10^10^ particles/mL	[63]
Mesenchymal stem cells (MSC)	2 × 10^6^ cells 10 mL CCM	UC	10 μg/mL	[64]

The initial centrifugations of a culture supernatant or a biological fluid at lower speeds allows, first, the removal of larger contaminants, generally at 300× *g* for 10 min, then of dead cells at 2000× *g* 10 min, and finally of cell debris at 10,000× *g* 30 min. In some cases, these passages are followed by supernatant filtering using a 0.2 μm syringe filter [57] to remove all particles larger than 200 nm, including residual apoptotic bodies and biological aggregates. These first pre-processing steps of culture medium are followed by proper exosomes isolation. Exosome pellet recovery is performed after treatments at a high speed (in a range from 100,000 to 120,000× *g*), resuspending it in phosphate-buffered saline (PBS). All the centrifugation steps are always performed at 4 °C to avoid the aggregation of proteins.

A valid alternative to standard UC is the density gradient UC (DG). In this case, the separation of exosomes is based on their size, mass, and density in medium with a progressively decreased density from the bottom to the top of the tube. Samples are layered as a narrow band on the top of the density gradient medium and are subjected to an extended round of UC [53]. A discontinuous iodixanol gradient consisting of 40% *w*/*v*, 20% *w*/*v*, 10% *w*/*v*, and 5% *w*/*v* solutions is prepared by diluting a stock solution of OptiPrep^TM^ in 0.25 M of sucrose/10 mM of Tris at pH 7.5. The gradient is set up in a polyallomer tube by the subsequent layering of 3 mL fractions of 40%, 20%, and 10% iodixanol solution, and 2.8 mL of 5% iodixanol solution. DG fractions of 1 mL each are collected from the top of the gradient and resuspended in PBS for a further 90 min of UC at 100,000 *g* [51].

The comparison of dUC, immunoaffinity, and OptiPrepTM DG revealed that this method isolated the pure population of exosomes from blood plasma [4]. It allowed the elimination of contaminants and enhanced the quality of EVs analysis [65], despite the fact that it may co-isolate EVs and certain lipoproteins [66]. However, DG is a laborious and time-consuming method. Unlike differential ultracentrifugation, a downside of density gradient ultracentrifugation is that its capacity is largely limited by the narrow load zone [67] density gradients.

## 7. Polymer-Based Precipitation

The isolation of exosomes by a polymer solution is relatively new, indeed, its principle was applied for the first time more than 50 years ago by Hebert [68] to isolate viruses. Prevalent results in exosome purification mainly ensue from the use of a less time-consuming commercial kit (ExoQuickt from System Bioscience or the Total Exosome Isolation Kit from Life Technologies). This method is based on the formation of a polymer network—generally consisting of polyethylene glycol (PEG) with a molecular weight of 8000 Da—that extracts water and forces less soluble components out of the solution. Although this method has a high scalability, it requires pre and post-cleanup. Indeed, the recovery of the precipitated sample is performed after incubation at 4 °C overnight, but the final collection in PBS [53,69] requires low-speed centrifugation or filtration.

A recent study from Chang et al. [70] presented a novel method for exosome isolation using Fe_3_O_4_ magnetic nanoparticles (MNPs) coated with polyethylene glycol (PEG). PEG chains can form reticular structures that allow the entrapping of proteins, aggregates, and impurities in the holes of MNP. Therefore, exosomes can be purified by removing the proteins using a permanent magnet. Unfortunately, the presence of residual magnetic material can cause the necessity of a time-consuming post-purification procedure.

Indeed, most studies [71] demonstrated that polymer-based precipitation is not a specific method because of the recovery of contaminants as protein aggregates, lipoproteins, and small cellular debris. Secondly, once isolated, the presence of the polymer material may not be compatible with downstream analyses. Nevertheless, the use of polymer-based precipitation may be appropriate to achieve an initial enrichment of exosomes, where the presence of contaminating non-exosomal materials can be problematic [72].

## 8. Size-Based Isolation Technique

The separation of exosomes based on size exclusion can be realized using ultrafiltration (UF) membranes and/or size exclusion chromatography (SEC).

UF allows the separation of exosomes from other soluble proteins and aggregates using matrices with defined molecular weight or size exclusion limits (Vivaspins^®^ or Amicons^®^). These vesicles can, for example, be selectively isolated based on a molecular weight greater than 200 kDa, followed by isolation with a diameter less than 200 nm [73,74].

UF is faster than UC and does not require special equipment. Furthermore, one or multistep concentrations can be efficient for large volume samples. However, a potential drawback could be the clogging and particle trapping due to the use of mechanical forces [53].

In SEC, the separation of exosomes is due to their small dimensions compared to other cellular debris and residual impurity or protein aggregates. A sample is loaded onto a packed column and passes through a selective porous resin: larger molecules are entrapped in the network structure while small molecules can pass faster through the pores and are eluted earlier [51]. Thus, in SEC separation, it is possible to underline several advantages such as the low contamination and high purity of the final sample, whose biological activity is preserved with superior reproducibility. Nevertheless, the procedure requires a long run time and it is not simple to scale up.

## 9. Microfluidic Technology for the Separation of the Exosomes

Although the development is still at an early stage, microfluidic technology is emerging as an efficient and rapid alternative to conventional isolation methods.

The techniques developed for microfluidic-based exosomal purification can be classified into two categories: chips with or without the application of external sources. The first ones allow an active sorting of the final sample due to the application of external sources such as an electric or magnetic field. Chips with no external forces achieve a passive sorting of nano-size objects through the integration of microfluidic components that drive exosomes into specific streamlines and immunoaffinity/size exclusion entrapping principles [75].

Immunoaffinity isolation exploits a specific interaction between characteristic surface proteins, membrane-bound antigens expressed by a specific subtype of EV, and immobilized antibodies. The isolation of the desired EV population could be obtained with an immuno-enrichment positive trapping or a negative selection of the unwanted exosome population (immuno-depletion) [76]. In 2010, Chen et al. demonstrated the rapid recovery of small EVs from both serum and conditioned culture medium with a microfluidic device containing antibody-coated surfaces [52].

The first example of size-based separation was reported by Davies et al. [77]. They sieved EVs directly from mouse blood through a pressure-driven filtration process on a membrane. Porous polymer monoliths were integrated into poly (methyl methacrylate) microfluidic chips as membranes with a proper size for the extraction of vesicles [77].

Wang and colleagues [78] designed a ciliated micropillar structure forming a microporous silicon nanowire. This nanowire-on-micropillar structure was able to trap particles selectively in the range of 40–100 nm. Silicon pillars were designed to have a distance too narrow (900 nm) to allow the passage of cells larger than 1 μm; at the same time, smaller cell debris can enter the micropillar area but is excluded by the ciliated nanostructure, which forms pores with diameters ranging between 30 and 200 nm in order to trap exosomes and small EVs selectively [79]. This method allows the selective sorting of vesicles with dimensions of less than 100 nm. However, despite the fact that the trapping step is relatively fast (10 min), the final recovery of the exosomal sample requires to dissolve the ciliated part of the silicon nanowire in PBS buffer overnight. Furthermore, active sorting mechanisms, such as acoustic separation and electromagnetic activation, could represent a valid alternative to usual microfluidics approaches. An acoustic nanofilter, as shown by Lee at al. [80] could use ultrasound standing waves to exert radiation forces on the biological fluid and allow the vesicles’ separation according to their mechanical properties such as their size and density.

All microfluidic technologies present important advantages compared to standard isolation techniques: they are not very time consuming, have high reproducibility, and allow the sorting of an EV subtype population. Nevertheless, generally, further off-chip additional steps for sample preparation are required, such as plasma extraction or reagent mixing [79], and these techniques are restricted to low sample volumes.

For this reason, all the mentioned techniques are not always mutually exclusive and it is possible to combine them or apply slight variations to the protocols within each group in order to overcome limitations in the purification processes.

It seems evident that technologies for the quality control and mass production of exosomes are desperately needed to achieve fast, highly efficient extraction procedures. Indeed, to overcome this invalidating drawback, recent studies seem to address more the molecular engineering of EVs.

Recently, Toledano Furman and Naama E. et al. developed a protocol for cell manipulation, allowing the formation of the so-called “nanoghost” [81] or “exosomes-mimetic” structures bypassing all the isolation steps in order to obtain a more reliable and versatile structure.

Nanoghosts reconstructed from the whole MSC cell membrane, in contrast to exosomes or other extracellular vesicles that are shed or bud from cells, are manufactured in a reproducible process by isolating intact MSC cell membranes (ghost cells) and extruding them into nanosized vesicles while entrapping a therapeutic of choice [82].

Moreover, also microfluidic technology allows through the extrusion method the recombination of the cell membrane surface to generate exosomes mimetics: nanovesicles contain mRNAs, intracellular proteins, and plasma membrane proteins and are shaped like cell-secreted exosomes [83,84].

## 10. Architectures of Exosomes and Their Biological Composition

An enormous heterogeneity characterizes the exosome architecture in terms of proteins, lipids, and genetic material, including messenger RNAs (mRNAs), microRNAs (miRNAs), other small non-coding RNAs, and genomic DNA (gDNA) expressed on the EV surface [85]. Regarding their biological composition, to date, a few studies have been conducted on specific cell lines highlighting this complex aspect. Among these studies, the highest cholesterol concentration (41–46%) was interestingly found in exosomes obtained from reticulocytes and human prostate cancer cells (PC-3). Despite the fact that exosomes secreted from oligodendrocytes only contain 2.2% cholesterol, they are highly enriched in phosphatidylcholine (40%), phosphatidylserine (25%), and phosphatidylethanolamine (20%). In contrast, 50% of the lipids found in B-cell-derived exosomes are ceramides [86].

The lipidomes of Huh7 exosomes showed a marked enriching of cardiolipins and lyso-derivatives (where one fatty acid tail is removed by hydrolysis) of phosphatidylserines, phosphatidylglycerols, and phosphatidylinositols. Meanwhile, lyso-phosphatidylethanolamines are rather enriched in U-87 MG exosomes. These lyso-derivatives are also enriched in Marrow Stromal Cell MVs but depleted from U-87 MG and Huh7 MVs [87]. Thus, the variety of the lipidic composition characterizes not only the EVs derived from different progenitor cells but also those derived from the same population.

Likewise, EVs’ proteome data suggests that the exosomal and MV proteomes of the same source-cell type are not directly comparable. Traditional exosome markers CD81 and CD9 are present both in MSC exosomes and MVs, but the level of enrichment seems to be higher in exosomes, while the PLP2 enrichment is unique to MVs.

Secondly, also the proteome profile of exosomes obtained from different source-cell types seems to be quite different. For example, exosomes isolated from cerebrospinal fluid (CSF) have specific proteins related to their tissue of origin. Tissue expression mapping according to the Database for Annotation, Visualization and Integrated Discovery (DAVID) knowledge base showed the presence of 373 brain-derived proteins; several markers of specific brain cell types, such as the typical microglia marker integrin alpha-M (ITGAM) and the receptor-type tyrosine-protein phosphatase C (PTPRC); and also neuron-specific markers such as enolase 2 (ENO2), dihydropyrimidinase-related protein 2 (DPYSL2), and vesicle-associated membrane protein 2 (VAMP2), which is a component of the soluble N-ethylmaleimide-sensitive factor attachment protein receptors (SNARE) complex [88].

Otherwise, Tsg101, PDCD6IP (Alix), and CD82 are only enriched in U-87 MG exosomes, while Flotillin-1 and tetraspanin-4 are highly enriched in all U-87 MG and Huh7 EVs [87]. Despite the variable composition, Immuno-Electron Microscopy localization studies, Western blot analysis, and the mapping of exosomal proteins have identified some common proteins located on the surface or in the lumen of nearly all exosomes (exosomal markers). Notably, exosomes are highly enriched in cytoplasmic proteins with various functions. Most exosomes contain MHC class I molecules and heat-shock proteins such as Hsp70 and Hsp90 as part of the stress response [89]. A significant number of proteins, such as tetraspanins (CD9, CD63, CD81) and Rab proteins (Rab11, Rab27a, Rab27b), are involved in their biogenesis process [90], while tubulin, actin, actin-binding proteins, and annexins proteins are responsible for membrane transport and fusion. Signal transduction and exosome release are instead mediated by protein kinases, heterotrimeric G-proteins, and molecules such as Alix and TSG101 [91].

Since exosomes have been discovered in almost all body fluids, including blood, urine, saliva, breast milk, cerebrospinal fluid, semen, amniotic fluid, and ascites [92], their specific profile (miRs, proteins, and lipids) can mirror the cellular origin and its physiological state as a “fingerprint” [93].

The application of exosomes in liquid biopsy could represent a valid alternative to traditional invasive methods in clinical diagnosis. However, although these markers are often enriched in specific exosomes, they are also present in EVs released by other progenitor cells.

## 11. Mechanism of Interaction in the Biological Environment

The rising interest in EVs is due to their capacity to induce phenotypic changes in acceptor cells [94]. Exosomes play a key role in the systemic propagation of patho-physiological mechanisms, including development, homeostasis, and immune surveillance/pathogen response [95]. EVs’ internalization and regulatory properties depend on factors such as the maturation, physiological and environmental conditions of target cells, or even on the vesicular proteomic and lipidomic profile generally determined by the progenitor cell type [96]. The fact that EVs’ cargo reflects their tissue of origin is relevant, since cancer cells are known to produce greater numbers of EVs containing signaling molecules compared to healthy cells.

First of all, tumor-derived EVs at hypoxic conditions stimulate the neo-vascularization and propagation of the angiogenic phenotype to endothelial cells [97]. EVs’ angiogenic cargo includes a wide range of molecules such as tissue factors, cytokines, tetraspanin, oncoproteins, sphingomyelin, and miRNAs [98]. As an example, a recent mass spectrometry analysis of GBM exosomes identified over 1000 proteins that exert angiogenic and tumor-invasive characteristics [98]. Pro-angiogenic factors include angiogenin, IL-6, IL-8, TIMP-1,and TIMP-2 that stimulate an angiogenic phenotype in normal brain endothelial cells and increase malignancy in a hypoxia-dependent manner [99].

Furthermore, tumor-derived EVs modulate the extracellular matrix through the proteolytic degradation of collagens, LNs, and fibronectin [100]. Matrix degradation has severe consequences on the tumor microenvironment, such as promoting host cell adhesion, motility, invasiveness, and apoptosis resistance. As an example, malignant ovarian ascites samples from patients with stage-I to -IV ovarian cancer contain matrix metalloproteases MMP-2-, MMP-9-, and uPA-loaded EVs with highly invasive properties [101]. Hallal et al. [102] reported increased podosome formation and extracellular matrix degradation in astrocytes cultured with GBM exosomes. Interestingly, this phenomenon seems to strongly correlate with tumorigenesis through decreased p53 levels. As consequence, EVs modify neighboring astrocytes to induce tumor-supportive functions and, moreover, drive peritumoral astrocytes to become tumorigenic themselves.

Conversely, in normal circumstances EVs are important in tissue homeostasis and organogenesis [103].

For example, platelet-derived EVs induce angiogenesis in vivo by facilitating the formation of endothelial capillaries [104]. Relevant studies show that exosomes produced by neurons, oligodendrocytes, astrocytes, and microglia have a key role in the protection and repair of brain tissue [105]. They could protect neurons by inhibiting neuronal apoptosis, modulate axon reconstruction and neurogenesis through vascular regeneration, and increase synaptic vesicle release [103]. For example, microglia-derived EVs can alleviate acute inflammatory responses by converting immature IL-1b into a biologically active molecule; regulate the excitation inhibition balance via the endocannabinoids content; and reduce the levels of amyloid-β, a neurotoxic peptide linked to Alzheimer’s disease [106].

Once released in the extracellular space, exosome internalization in a recipient cell occurs via two different mechanisms: direct interaction resulting in EV fusion with PM or endocytic uptake.

Endocytosis seems the principal pathway. It can involve multiple routes: the clathrin-dependent or independent pathway, the caveolin-mediated mechanism, micropinocytosis, phagocytosis, or lipid raft-mediated uptake [107]. Lipid composition is heavily involved in intracellular trafficking. The phosphatidylserine enrichment of oligodendrocyte-derived exosomes activated pinocytosis in a subset of microglia macrophages without antigen-presenting capability [108]. Additionally, the sphingolipids within the EV have an important role in binding and endocytosis, possibly through cholesterol-rich microdomains in dendritic cells [107]. Additionally, surface and cytoplasmic proteins anchored to the vesicle lipid bilayer membrane are involved in specific ligand-receptor type interactions. They include tetraspanins, TNF, TRAIL, FasL, integrins, or T cell immunoglobulin [109]. For example, tetraspanins are highly abundant on exosomes’ surfaces and notably have been shown to be involved in a number of processes, including vesicular and cellular fusion. The treatment of recipient cells with antibodies against the tetraspanins CD81 or CD9 can reduce the uptake of EVs by dendritic cells [110]. Cells over-expressing Tspan8 released EVs bearing a Tspan8-CD49d complex, the presence of which contributed to EV uptake by rat aortic endothelial cells [111].

Although there are several types of proteins capable of interacting specifically with a cellular target, none have been established as sufficient and necessary for EV internalization. Many EV subtypes share common surface proteins, and it is possible that one of them acts as a general ligand for a receptor, enabling vesicle internalization.

One unresolved question currently vexing the EV field is whether EV uptake is a cell type–specific process or whether the process is unspecific. The mechanisms of EV uptake into the cytosol of the recipient cell are still unclear and seem to act both in a generic and a specific manner.

## 12. Applications of Exosomes for the Diagnosis and the Therapy of Gliomas

In the last few decades, evidence about the role that exosomes secreted by healthy and tumor cells have in the growth and spread of such a complex environment suggested their use as a diagnostic and prognostic indicator of tumor progression [88], even for brain malignancies such as glioma (Table 2).

Unmodified exosomes can cross BBB thanks to their small size, flexibility, and the presence of adhesive proteins on their surface, while their endogenous origin and the presence of a cellular lipidic bilayer minimize immunogenicity and toxicity, supporting their stabilization in blood circulation [112]. Furthermore, the application of exosomes both as diagnostic and therapeutic tools is deeply correlated to their long in vivo blood circulation and biodistribution [113]. Exogenous blood circulating exosomes are enriched in proteins and genetic material, which should allow an earlier and more accurate diagnosis. On the other hand, EVs artificially introduced into circulation with a prolonged half-life at the target site could achieve a lower and more efficient therapeutic dosage of the active substance carried. Unfortunately, to the best of our knowledge, exosome in vivo biodistribution studies seem to be very controversial. Establishing a unique biodistribution mechanism seems to be impossible due to the numerous variables involved; the route of administration, the progress of the disease, the exosomal parent cell source, as well as the different target cell types available to internalize the circulating EVs are just the main parameters to keep in consideration.

Direct intravenous, intraperitoneal, or subcutaneous injection of breast 4T1, MCF-7, and prostatic PC3 tumoral exosomes, for example, result in rapid clearance from the blood circulation and accumulation in the liver, spleen, lung, and gastrointestinal tract [56]. The intravenous injection of blood cell-derived EVs showed an uptake by the liver (44.9%), bone (22.5%), skin (9.7%), muscle (5.8%), spleen (3.4%), kidney (2.7%), and lung (1.8%) [114]. In contrast, B16 melanoma-derived EVs were mainly taken up by lungs and spleen [115]. Regardless of the delivery route and cell source, the half-life of the majority of systemically injected exosomes seems often to be very short due to the macrophage uptake in the reticuloendothelial system [116], and this could lead to a rapid clearance. However, their hemodynamic is still debated. Indeed, differently derived exosomes exhibit different circulation, biodistribution, and clearance properties compared to their normal counterparts, with additional changes associated with tumor progression and response to treatment [115].

As for traditional nanovectors, it seems clear that even for exosomes, the engineering of nanovesicular structures through post-isolation modifications can be helpful for diagnostic and moreover therapeutic application [117]. For example, in addition to naturally expressed protein, the conjugation of specific targeting ligands, such as antibodies and peptides, may enable specific interactions with target cells. Further advantages can be achieved through the pre- or post-isolation loading of unmodified EVs with molecules of interest [118].

**Table 2:** Summary of the literature assessing exosomes as a drug delivery system and diagnostic biomarkers in in vitro and in vivo glioma models.

Cell Source	Cargo	Application	Models	Reference
Raw264.7	SPIONs/Curcumin/RGE peptide	Imaging and anti-tumor therapy	In vitro (U251) In vivo glioma mice model xenograft	[62]
MSC	miRNA-584	Anti-tumor miRNA therapy Inhibition glioma growth	In vitro (U-87 MG) In vivo U-87 MG xenograft nude mouse mode	[119]
MSC	miR-199	Inhibition glioma growth Chemosensitivity	In vitro (U251) Ex vivo immunohistochemistry tumor-bearing nude mice	[120]
MSC	miR-146b	Anti-tumor miRNA therapy Inhibition glioma growth	In vitro (9L glioma) Ex vivo rodent model (9L glioma) xenograft	[64]
U-87 MG X12 cells	miR-1	Anti-tumor miRNA therapy Inhibition glioma growth	In vitro (U87 and X12 GBM) In vivo xenograft nude mouse model	[121]
MSC	anti-miR-9	Chemosensitivity	In vitro (U-87 MG T98G)	[122]
U-87 MG	PTX/DXR	Delivery anticancer drugs	In vitro (U-87 MG) In vivo brain imaging of embryos zebrafish model	[123]
Brain endothelial cell (bEND.3)				
Mouse fibroblast cell line (L929)	KLA peptide LDL/MTX	Delivery of anticancer drug and therapeutic targeted peptides	In vitro (U-87 MG) In vivo glioma mice xenograft	[18]
MSC	miR-124	Anti-tumor miRNA therapy Dysregulation of cellular metabolism	In vitro (GSC26-28 GSC6-27) In vivo glioma mice xenograft	[124]
Natural killer-92MI	-	Immunotherapy Inhibition Glioblastoma growth	In vitro (U-87 MG) In vivo glioma mice xenograft	[125]
CSF	miR-21	Diagnostic biomarker	-	[126]
Serum	miR-21/miR-222/miR-124-3p	Diagnostic biomarker	-	[127]
CSF	miR-21 miR-103, miR-24, and miR-125	Diagnostic biomarker	-	[128]
Serum	miR-320/miR-574-3p/RNU6-1	Diagnostic biomarker Tumorigenesis factors	-	[129]
Serum	miR-301a	Diagnostic biomarker	In vitro (H4)	[130]
T98G cell line	L1CAM	Tumorigenesis factor	Chick embryo brain tumor model	[131]
Plasma	CAV1 IL-8	Hypoxia-induced, proangiogenic proteins	In vivo glioma mice xenograft	[132]
Blood	EGFR/EGFRvIII	Diagnostic biomarker	μNMR	[133]
Blood	PTRF	Diagnostic biomarker	In vitro (LN229 U-87 MG U251) In vivo mouse model xenograft	[134]

## 13. Exosomes as Biomarker in the Glioma Diagnosis

An ideal biomarker should allow the early detection of disease and potentially differentiate the progression of physiological states predicting response to therapy. To date, several studies have focused on the entire circulating pool of biomarkers but, interestingly, the EVs derived from fluid biopsies should represent a new approach for personalized medicine. Indeed, exosomes originating in cancer cells induce tumor-promotion effects, releasing principally proteins such as growth factor and oncoproteins, as well as mRNAs, miRNAs, and siRNA, which mediate specific signaling machinery related to dysregulated cell growth [135]. Therefore, it seems clear that, compared to a traditional diagnostic marker, exosomes should enhance, through a noninvasive approach, the synergic diagnostic activity of combined biomolecules [136]. Furthermore, their genetic and proteomic content should reflect the status of parental tumor cells and allow the identification of rapid changes in the tumor cell state and progress [137].

### 13.1. Single and Combined miRNAs Model as Glioma Diagnostic Tools

To date, miRNAs seem to have taken the lead in research as the most potential non-invasive diagnostic glioma biomarkers [137,138,139,140,141,142,143,144,145] (Table 3). For this reason, a great deal of effort has been spent on the establishment of exosomal miRNAs as glioma biomarkers.

First of all, accumulating evidence suggests the importance of the miR-21 levels in body fluids, in particular CSF, as indicators of the poor prognosis of various cancer types, including gliomas. Shi et al. [126] tested the serum and CSF exosomal miR-21 of 70 glioma patients with recurrence. Notably, the serum EV levels of miR-21 were unaltered, while CSF-EVs seem to be an efficient primary indicator of glioma recurrence. This finding was further substantiated by Akers et al. [138]; the miR-21 levels in GBM EVs patients were found to be around 10-fold higher than in non-tumor CSF–EVs, as was also confirmed in a more extensive cohort study where the CSF EV miR-21 levels show a high sensitivity (87%) and specificity (93%). Despite these encouraging results, miR-21 is abundantly expressed, and its upregulation is associated with numerous types of cancer [139] and numerous pathological pathways, such as immune cell activation and death receptor-mediated intrinsic apoptosis. Thus, the concurrent expression of several miRNAs in a specific glioma grading may allow a more precise diagnostic and prognostic outcome. Next-generation sequencing, microarray, and digital PCR have been recommended as good downstream analytical platforms for exosome miRNA quantification in order to further assist the development of translational medicine [140]. qRT-PCR should give information about the distribution of the miRNA pattern in different EV subpopulations, as demonstrated in the CSF-EVs of GBM patients [128] highly enriched in miR-21, as expected, but also miR-103, miR-24, and miR-125.

Santangelo et al. [127] analyzed the synergic activity of exosome-associated miRNA. They demonstrated that the miR-21, 222, and 124-3p combination increases the diagnostic efficiency for distinguishing High-Grade Glioma (HGG) patients from healthy controls and Low-Grade Gliomas (LGG), predicting glioma grading before surgery. Remarkably, they were able to correlate specifically the miR-21, 222, and 124-3p expression values to HGG (grade III gliomas and GBM IV) compared to healthy controls and LGG (grade I–II gliomas). Indeed, despite the fact that the miR-222 expression was significantly higher in LGG than that found in healthy controls and that miR-21 can also be observed in malignant brain tumors of non-glial origin, the expression values of miR-21 and 124-3p in LGG were very similar to those detected in healthy controls. To control GBM heterogeneity, also the role of siRNA was investigated, with a particular focus on RNU6-1 [141]. Notably, RNU6-1 is synthesized by RNA polymerase III and negatively regulated by tumor suppressors as a retinoblastoma protein (Rb) and a phosphatase and tensin homolog (PTEN). These pathway dysfunctions are evident in GBM progression, but, unfortunately, not statistically relevant studies on a control group of patients with other nervous system disorders or other brain tumors were conducted to provide complete information on selectivity in GBM neuroimaging.

Lan et al. [130] reported that exosomal miR-301a may serve as an independent prognostic indicator via the activation of the AKT and FAK signaling pathways through the downregulation of PTEN. Serum exosomal miR-301a extracted from grade IV GBM patients were significantly up-regulated compared to healthy controls, promoted the proliferation and invasion of glioma-derived H4 cells, and was additionally correlated with ascending pathological grades.

It seems evident that exosomal miRNA profiles are different from formerly described “free-circulating” miRNAs in GBM patients and appear to be superior for diagnostic purposes. Nevertheless, glioma represents an extremely complex and heterogeneous microenvironment. Thus, the identification of reliable and specific biomarkers is still challenging.

The association of different biomarkers seems to confirm, also in this case, the control of disease heterogeneity. Indeed, Manterola et al. [129] used serum from 75 patients with newly diagnosed GBM. As expected, they found that RNU6-1 was consistently an independent predictor of a GBM diagnosis, but even more its expression levels in association with 2 microRNAs (miR-320 and miR-574-3p) were significantly high in GBM patients.

### 13.2. Exosomal Protein and Tumorigenic Factors as Glioma Biomarkers

As for miRNA biomarkers, exosomal proteins show a greater specificity in comparison with free serum proteins. It was found that many exosomal proteins such as CLIC1 [142], CAV 1 [143], IL-8 [144], immuno-globulin (Ig) G2 and IgG4 [145], VEGF, and EGFR play a key role in the growth and dissemination of tumors. An example, immunoglobulin superfamily protein L1CAM (L1, CD171) normally facilitates neuronal migration, differentiation, and axon guidance during development [131]. Exosomes isolated from the conditioned media of the human T98G GBM cell line seem to overexpress the L1 domain on their surface. This results in increased cell motility, proliferation, and invasiveness in the three human glioma cells tested (T98G/shL1, U-118 MG, and primary GBM).

Chloride Intracellular Channel protein CLIC1 is involved in the cell cycle progression and chemoresistance activity in a variety of different tumoral environments, including GBM [142]. Notably, the exosomes released by CLIC1-overexpressing GBM cells accelerate the in vitro cell proliferation and tumor engraftment in vivo. However, the modulation of CLIC1 protein expression in GBM cells induces phenotypic changes only on molecular cargo, since no relevant modification was observed on the EV secretion or uptake mechanism.

Furthermore, the proteomic profile of exosome could also reflect the oxygenation status of GBM cells and patient tumors. Hypoxic GBM cells secrete exosomes enriched in several proteins implicated in tumor aggressiveness. IL8 and CAV1 are reported [132] as well-established hypoxia-responsive factor, and have been suggested to have a role in the development of aggressive gliomas. CAV1 showed a corresponding enrichment in hypoxic compared with normoxic exosomes in vitro and in GBM patients compared with control exosomes; IL8 was found in quantity 3.4-fold higher in GBM tumor-bearing mice compared with control mice and accumulated in the hypoxic regions of GBM xenografts.

Treps et al. [146] demonstrated that exosomes secreted from ex vivo cultured Glioblastoma Stem-like Cells (GSCs) derived from primary GBM patients carry higher levels of VEGF-A, as compared to healthy donors. However, VEGF-A does not allow the detection of glioma grading; moreover, its expression in numerous tumor cells and tumor vasculature does not assure that the EV-trapped VEGF-A found in plasma arises selectively from GSCs.

EGFR and its active mutant EGFRvIII have a high pro-tumorigenic function that confers a growth advantage to GBM. Elevated levels of EGFR and EGFRvIII were found to be detectable in both serum and CSF exosomes with a high diagnostic sensitivity [133]. However, numerus clinical trials have demonstrated that the EGFRvIII levels did not allow one to distinguish between low- and high-grade gliomas. Interestingly, Huang et al. [134] showed that EGFRvIII overexpression increased the polymerase I and transcript release factor (PTRF) expression. For this reason, they explored PTRF’s ability as biomarker: firstly, they proved PTRF’s presence on exosomes’ surfaces and the capability of improving EV secretion and GBM cell proliferation; secondly, they analyzed the PTRF/CD63 ratio in tumor samples and relative donated blood from 18 WHO grade II and grade IV samples, finding that a higher PTRF/CD63 ratio at the protein level was detected in WHO grade IV samples in both tumor tissues and serum exosomes, indicating a more selective prognosis.

It seems clear that, as our knowledge of the activity of tumor-derived exosomes improves, we can better identify all the genetic and proteomic content that mirrors the real status of glioma progression.

Exploiting the diagnostic potential of exosomes requires a thorough understanding of their composition at both the individual and population levels. To date, both miRNA and growth factor seem to be extremely variable, and this heterogeneity is present at different levels. The type and the quantity of overexpressed biomarkers are related to the biological fluid of extraction, exosome subtyping, glioma grading, and patient profile. Actually, engineering nanovesicles simultaneously with different biomarkers seems to be the best approach for a highly predictive diagnosis. Nevertheless, as we have seen, these biomarkers are often correlated with other neurodegenerative diseases or identify an advanced stage of glioma. Thus, further research, especially with respect to gliomagenesis, has to be conducted.

**Table 3:** Deregulated miRNAs from different sample types as potential diagnostic biomarker in glioma.

miRNA	Cancer Type	Regulation	Patient Source	Reference
miR-21	Glioma (grade I to IV)	Up	Plasma	[147]
miR-15	Glioma (grade III to IV)	Up	Tissue/cell	[147]
miR-221/222	Glioma (grade III to IV)	Up	Plasma	[148]
miR-210	Glioma (grade I to IV)	Up	Serum	[149]
miR-29	Glioma (grade I to IV)	Down	Serum	[150]
miR-181a/b/c	GBM	Down	Tissue	[151]
miR-497, miR-125b	GBM	Down	Serum	[152]
miR-15b-5p, miR-16-5p, miR-19a-3p, miR-19b-3p, miR-20a-5p, miR-106a-5p, miR-130-3p, miR-181b-5p, miR-208a-3p	Astrocytoma (grade II to IV)	Up	Serum	[153]

## 14. Exosomes in the Treatment of Glioma

Exosomes’ ability as signaling in local and remote intercellular crosstalk enables them to deliver more efficiently macromolecular drugs, lipids, proteins, and genetic material such as miRNA siRNA to the brain [154].

Further benefits can be achieved through exogenous or endogenous modification strategies.

Exogenous modification occurs after cell culture production through the surface conjugation of specific receptors or encapsulation with hydrophilic/hydrophobic compounds. Exosomes might be modified endogenously through manipulation at the cellular level. In this case, the modification of progenitor cells can occur by the incubation of drug molecules or by transfection or transduction with expression vectors that lead to the secretion of EVs containing drug molecules, viral proteins, nucleic acids, RNA, and proteins [117].

### 14.1. miRNAs Regulation as a Therapeutic Strategy

At date, exosomes derived from MSCs transfected with anti-tumor miRNAs have been found to be promising therapeutic tools for glioma therapy [155].

An example, Kim et al. [119] used exosome derived from MSCs transfected with miRNA-584. miRNA-584 acts as a tumor suppressor in some cancers and inhibits specifically glioma cells activity by binding to the 3′-UTR of CYP2J2. Interestingly, they demonstrated that MSCs exosomal miRNA-584 affects invasive ability of U-87 MG cells in vitro and decrease tumor mass weights in U87 MG xenograft nude mouse model.

The glioma development could also be prevented by down-regulation of Arf GTPase- activating protein-2 (AGAP2), a target gene of microRNA-199a (miR-199a). In lines with this finding, Yu et al. [120] showed that miR-199a when delivered via MSCs-derived exosomes inhibiting in vitro U251 glioma cell proliferation, migration and invasion. Additionally, Katakowski et al. [64] tested MSC exosomes as a miRNA delivery vehicle in malignant glioma. Over-expressed miR-146b in MSC exosomes (M146-exo) were tested both in vitro and in vivo. Initially, they conducted an in vitro study on 9L cells and found out that, after 7 days, in vitro growth of M146-exotreated 9L cells was significantly less than normal MSC exosomes-treated control. Finally, to determine if M146-Exo had an anti-tumor effect in vivo, they administered M146-exo or M67-exo to Fischer rats bearing 9L gliosarcoma. One intra-tumor injection of M146-exo, 5 days after intracranial tumor xenograft implantation, led to a significant reduction in tumor volume at 10 days post-implant compared to control.

Although MSC exosomes are the most commonly used therapeutic tool for miRNA transfection in glioma treatment, also tumoral exosomes could be applied. Indeed, it can not be excluded that the overexpression of specific tumoral biomolecules may further favor cellular exosomes communication with receiving cell and selectivity to the tumoral microenvironment. In this regard, Bronisz et al. [121] identified miR-1 deficiency as a contributor to glioma invasiveness and neovascularization and demonstrated that reintroduction of miR-1 into GBM exosomes through transfection of U-87 MG and X12 cells reverted paracrine-stimulated malignancy and microenvironmental remodeling by tumor. These findings support the hypothesis that miRNA replacement approaches have strong therapeutic potential and can be mediated by extracellular vesicles. In addition, they raise the possibility that modified tumor exosomes might be employed as biological Trojan horses to suppress tumor cells and their effect upon the brain microenvironment.

Reinforcing this point, it seems clear that miRNAs are often involved in the inhibition of tumor developmental processes. On the other hand, miRNA can behave not exclusively as tumor suppressors but also as oncogenes. Indeed, deregulation of microRNA expression has been observed in several cancers’ progression mechanisms, including GBM.

Among these, Munoz et al. [122] focused on miR-9 molecules that have been shown to suppress the mesenchymal differentiation of GBM cells. They identified an increase of miR-9 concentration in TMZ-resistant GBM cells, involved in the expression of the drug efflux transporter P-glycoprotein. On this basis, they showed that reversed chemoresistance of GBM cells to TMZ occurred by targeting of anti-miR through MSCs. To block miR-9, they tested anti-miR-9-Exosome obtained from transfection with human bone marrow–derived MSC. Cell viability assay showed that the anti-miR-9-exosomes treatment enhances TMZ-induced cell death in U87 MG and T98G.

Furthermore, transwell studies indicated that MSCs could communicate with cancer cells through gap junctional intercellular communication (GJIC) and also through secreted exosomes. Although further investigations have to be performed, this finding is relevant in the comprehension of exosomes and in vivo studies could confirm that the release of exosomes can affect GBM at a considerable distance from the MSCs.

### 14.2. Exosomes as Drug Delivery Systems

Finally, drug-loaded exosomes as drug delivery systems have been investigated by several authors. Although cellular packaging during EV biogenesis is a common and simple strategy, it involves the use of large amount of material and often has inefficient loading outcomes. The loading of EVs with therapeutic products after their isolation could represent a valid alternative. The simplest method is the passive incubation of isolated EVs with the therapeutic molecule, as reported by Yang et al. [123].

First of all, they highlighted the effect of the molecular characteristics of exosomes derived from the human brain neuronal glioblastoma-astrocytoma U-87 MG and the brain endothelial bEND.3 on their ability of interaction and the crossing of biological barriers. Their results demonstrated that bEND.3-derived exosomes allowed a higher internalization of the fluorescent marker in bEND.3 cells via an energy-dependent internalization process (cell uptake studies were performed both at 37 °C and 4 °C). Moreover, this active process was assumed to be receptor-mediated endocytosis by CD63 tetraspanins transmembrane proteins that are overexpressed in brain endothelial cells. Reinforcing this point, they reported the use of both U-87 MG and bEND.3 exosomes to deliver paclitaxel (PTX) or doxorubicin (DXR) across the BBB in a zebrafish model of brain tumor employing U-87 MG glioma. Freely administered DXR and PTX are not able to cross the BBB while the vesicles-packaged tool facilitated drug delivery across the BBB, reducing tumor progression.

Promising experiments showed the possibility of simultaneous exosome engineering through surface modification and drug loading for imaging and therapy in vitro and in vivo. Jia et al. [62] firstly loaded superparamagnetic iron oxide nanoparticles (SPIONs) and curcumin (Cur) into exosomes and then conjugated the exosome membrane with neuropilin-1-targeted peptide (RGERPPR, RGE) by click chemistry to obtain glioma-targeting exosomes with imaging and therapeutic functions.

Furthermore, the engineering of exosomes by both drug loading and surface functionalization was also recently performed by Ye et al. [18]. They reported a double functionalization of methotrexate (MTX)-loaded EVs with both the targeting pro-apoptotic peptide, KLA, and the targeted low-density lipoprotein, LDL, for selective binding to the LDL receptor (LDLR) overexpressed on the BBB and GBM cell lines. Indeed, the role of KLA was highlighted by observation under confocal microscopy. EVs decorated with KLA and LDL (EVs-KLA-LDL) were incubated with U-87 MG glioma spheroids for 12 h to assess their penetrating ability. EVs modified with the targeting peptide had an increased uptake by U-87 MG cells as well as an augmented permeation capacity into tumor cells. Furthermore, ex vivo fluorescence studies of the brain performed after intravenous injections of DiR-labeled EVs or EVs-KLA-LDL confirmed that EVs-KLA-LDL crosses the BBB and penetrates the brain more efficiently than blank-EVs, which might be attributed to the interaction between the LDL peptide and the LDLR over-expressed at the BBB.

Thus, the engineering of the EV surface prompts the process of membrane receptor-mediated internalization both in vitro and in vivo and provides a unique opportunity to deliver KLA and MTX to the U-87 MG glioma. To improve the BBB permeation, studies have focused not only on chemical modifications and genetic engineering. The application of a focused ultrasound system (FUS) produce a reversible and local disruption of BBB; Bai et al. [156] designed a drug delivery system that combines doxorubicin (Dox)-loaded Exos derived from macrophages (R-Exos) and blood serum (B-Exos) for glioma diagnostics and therapy with two FUS treatments. Importantly, through this combination, they demonstrated a visible regression of tumor growth in orthotopic gliomas and an extended survival time, leading to a significant improvement over free Dox and Exos-Dox treatments.

## 15. Conclusions

Since the identification of EVs as exogenous intercellular communication tools, in the last few years the field of exosomes-based drug delivery has greatly expanded because of their interesting properties:

- nanosized and specific compositions minimize recognition by the mononuclear phagocyte system;
- patient self-derived nature eludes immune system activation;
- low immunogenicity potentially delivers exosomes in a cell type-specific manner;
- surface composed of GM3, sphingomyelin, and cholesterol supports the stabilization of the vesicles in the blood circulation and stimulates membrane fusion;
- surface proteins have likewise been linked to membrane fusion in cell–cell and virus–cell interactions;
- proteo-lipid architecture protects the encapsulated cargo.

Moreover, additional targeted functions have been developed arising from the engineering of the donor cells or the post-isolation modification of exosomes’ surfaces, always preserving their inherent properties. Furthermore, as exosomes are structures encased in a lipid bilayer membrane with an aqueous core, they are capable of housing both hydrophilic and lipophilic drugs.

Despite early promising experimental results, the application of exosomes for therapeutic drug delivery—in particular, for the treatment of primary brain tumors—is far from a clinical translation. First, a major limitation is the lack of standardized techniques for the isolation of exosomes because of time-consuming procedures, poor reproducibility, and low production yield. Notably, protein aggregates and other cell debris can also affect exosomes’ purity. Once extracted from biological fluids, several distinct approaches could be applied for the loading of exosomal carriers with therapeutic cargo. However, it is worth noting that neither of these strategies is a satisfactory, scalable, and cost- effective procedure for the efficient encapsulation of nanovesicles.

Key to the development of exosomes is also a better understanding of their molecular composition in order to overcome the problems correlated to their high heterogeneity. Future technical advances may lead to significant progress in the identification of a complete proteome and lipidome profile to distinguish each exosome subtype. Additionally, a few studies have reported information about the clearance and biodistribution toxicity profiles of exosomes, and they seem to be quite controversial.

The elevated levels of signal transduction, migration, adhesion, and antigen proteins incorporated into the lipid membrane of exosomes, compared with proteins of various other biological functions, suggest that exosomes may be important for cellular communication both locally between neighboring cells and to distant cellular targets with a high stability and specificity to a targeted tissue. Conversely, some evidence about rapid clearance and uptake by the reticuloendothelial system of unmodified exosomes suggests that their primary function is not likely to be communicating with distant cellular targets but possibly exchanging information with neighboring cells and cells of the immune system, limiting their accumulation, especially in brain tumor tissue. In conclusion, the use of exosomes for the treatment of brain tumors is far from clinical reality. Can the exosomes secreted by tumor cells be utilized for the detection of tumor progression?

May exosomes switch their contents and role from a physiological to a pathological one during cancer progression? Are they able to modify the surrounding microenvironment?

Once administrated, how far exosomes can travel in the body, and how long they can maintain stability?

Substantial investigations about their uptake and key mechanisms in their biological environment, pharmacokinetics, pharmacodynamics, and toxicity profiles should be conducted to obtain a reliable and stable delivery system and prevent potential side effects.
